# Minimally Invasive Surgery in Gastric Cancer

**DOI:** 10.3390/cancers18121876

**Published:** 2026-06-09

**Authors:** Jane Chungyoon Kim, Hyuk-Joon Lee

**Affiliations:** 1 Department of Surgery, Seoul National University Hospital, Seoul 03080, Republic of Korea; janeckim88@snu.ac.kr; 2 Department of Surgery, Seoul National University College of Medicine, Seoul 03080, Republic of Korea; 3 Cancer Research Institute, Seoul National University College of Medicine, Seoul 03080, Republic of Korea

**Keywords:** gastric cancer, minimally invasive surgery, laparoscopic gastrectomy, robotic gastrectomy, reduced-port surgery, surgical outcomes, learning curve, centralization, quality control

## Abstract

Minimally invasive gastrectomy has become an established surgical approach for gastric cancer, supported by randomized trials demonstrating oncologic outcomes comparable to open surgery. Initially applied mainly to early-stage disease, laparoscopic gastrectomy has gradually expanded to selected patients with advanced gastric cancer and to more complex procedures, including total gastrectomy. In parallel, robotic surgery and reduced-port techniques have further broadened the technical spectrum of minimally invasive gastric cancer surgery. This review summarizes current evidence on laparoscopic, robotic, and reduced-port gastrectomy, with emphasis on randomized trials, large-scale clinical studies, and recent guideline recommendations. Laparoscopic distal gastrectomy has shown robust oncologic equivalence in early and selected advanced gastric cancer, while evidence for laparoscopic total gastrectomy continues to grow, particularly in experienced centers. Robotic and reduced-port approaches appear feasible and safe in selected settings, although clear superiority over standard laparoscopy has not been established. As minimally invasive techniques are adopted more widely, careful consideration is needed during program development and expansion. Patient selection, surgeon experience, learning curve, institutional volume, standardized operative procedures, quality control, and multidisciplinary support are important factors for safe implementation. Future studies should focus not only on comparing surgical approaches, but also on defining how minimally invasive gastrectomy can be applied safely and consistently across diverse clinical settings.

## Simple Summary

Minimally invasive surgery is widely used to treat gastric cancer as it can reduce surgical trauma while maintaining similar cancer outcomes to open surgery. Its use has expanded from early-stage disease to more complex procedures, including total gastrectomy, with the development of robotic and reduced-port techniques. This review summarizes current evidence and discusses how these approaches can be applied in practice. When introducing or expanding minimally invasive gastrectomy, factors such as patient selection, surgeon experience, learning curve, hospital volume, and quality control should be considered to ensure safe and effective treatment.

## 1. Introduction

Gastric cancer remains a major global health burden, and surgical resection with adequate lymphadenectomy continues to be the cornerstone of curative treatment [1]. Over the past three decades, minimally invasive approaches have been increasingly applied to gastric cancer surgery, with the aim of reducing surgical trauma while maintaining oncologic principles.

Laparoscopic gastrectomy was initially introduced for early-stage disease in highly selected patients. Early clinical experiences suggested potential benefits in perioperative recovery, but concerns were raised regarding the adequacy of lymph node dissection, margin status, and technical reproducibility [2]. To address these issues, multiple prospective randomized trials were conducted, primarily in East Asia, evaluating the safety and oncologic outcomes of minimally invasive surgery in comparison with open gastrectomy.

Accumulating evidence from these studies has supported the use of laparoscopic gastrectomy in early-stage gastric cancer and has gradually extended its indications to selected patients with advanced disease [3,4]. In Korea, the Korean Laparoendoscopic Gastrointestinal Surgery Study Group (KLASS) conducted a series of multicenter trials evaluating laparoscopic gastrectomy in different clinical settings. Evidence from Japanese and Chinese randomized trials has also supported the oncologic safety and feasibility of minimally invasive gastrectomy, while Western studies have provided important perspectives on its application in different healthcare settings.

In addition, further technical developments, including laparoscopic total gastrectomy, robotic gastrectomy, and reduced-port approaches, have expanded the scope of minimally invasive techniques. However, the application of minimally invasive gastrectomy in routine clinical practice remains variable. Differences in institutional volume, surgical expertise, perioperative management, and availability of structured training and quality-control systems may influence outcomes, particularly in more complex procedures such as D2 lymphadenectomy and total gastrectomy.

In this narrative review, we summarize current evidence on curative-intent minimally invasive gastrectomy, including laparoscopic, robotic, and reduced-port approaches. Relevant studies were identified through PubMed/MEDLINE searches and manual screening of references from major trials, guidelines, and review articles, with emphasis on randomized controlled trials, prospective multicenter studies, and selected large retrospective or registry-based studies published up to 2026. We also discuss factors relevant to interpreting and implementing these findings in clinical practice.

## 2. Evidence for Laparoscopic Gastrectomy

### 2.1. Laparoscopic Distal Gastrectomy for Early Gastric Cancer

Laparoscopic gastrectomy for gastric cancer was first introduced by Kitano and colleagues in 1994, who reported a laparoscopic-assisted Billroth-I distal gastrectomy for early gastric cancer [5]. This initial experience marked the conceptual shift from open radical gastrectomy toward a minimally invasive approach that aimed to preserve oncologic principles while reducing surgical trauma. In the years that followed, a growing body of retrospective and single-center prospective studies suggested that laparoscopic distal gastrectomy showed comparable oncologic outcomes to open surgery in carefully selected patients with early gastric cancer [6,7,8,9]. These early observations provided the rationale for subsequent prospective randomized validation.

This culminated in several large randomized controlled trials from East Asia. The phase III multicenter KLASS-01 trial in Korea randomized 1416 patients with clinical stage I gastric cancer to laparoscopic (LDG) or open distal gastrectomy (ODG) across 13 high-volume centers [10]. The primary end point, 5-year overall survival, was comparable between the two groups (94.2% vs. 93.3%) [11]. Gastric cancer-specific survival and recurrence patterns were also similar, with no differences in locoregional, hematogenous, or peritoneal recurrence. Notably, LDG was associated with a significantly lower rate of early postoperative complication compared with ODG (13.0% vs 19.9%, *p* = 0.001), largely driven by fewer wound-related events, including surgical site infection and dehiscence (3.6% vs. 7.0%, *p* = 0.005) [12]. In addition, LDG resulted in lower intraoperative blood loss and shorter hospital stay, although operative time tended to be longer than in the open surgery group.

Consistent findings were reported in the Japanese Clinical Oncology Group trial JCOG0912, a phase III non-inferiority randomized controlled trial comparing LDG and ODG in patients with clinical stage I gastric cancer [13]. A total of 921 patients were enrolled and randomized across 33 institutions in Japan. The primary endpoint, 5-year relapse-free survival, was comparable between the two groups (95.1% in the LDG group and 94.0% in the ODG group), confirming non-inferiority of the laparoscopic approach [14]. However, rates of in-hospital grade 3–4 surgical complications were similar between groups, with no significant difference observed (3.3% in LADG vs. 3.7% in ODG, *p* = 0.72) [15].

Collectively, these trials provided the first high-level evidence supporting the feasibility and safety of laparoscopic distal gastrectomy for early-stage disease, establishing oncologic noninferiority compared with open surgery. It should be noted that most early randomized trials evaluated laparoscopy-assisted distal gastrectomy, the prevailing minimally invasive approach at that time, in which reconstruction was generally performed extracorporeally through a mini-laparotomy rather than intracorporeally as in totally laparoscopic distal gastrectomy. Postoperative morbidity outcomes were not uniform across trials: KLASS-01 showed fewer overall complications after LDG, whereas JCOG0912 reported similar major complication rates between LDG and ODG. Despite variability in morbidity outcomes, selected short-term recovery parameters favored laparoscopic surgery, such as reduced blood loss, earlier bowel recovery, reduced analgesic use, and shorter hospital stay. Formal quality-of-life outcomes were not extensively addressed in the main reports. These findings support the use of laparoscopic distal gastrectomy as a standard approach for early gastric cancer in appropriately selected patients.

### 2.2. Laparoscopic Distal Gastrectomy for Advanced Gastric Cancer

While the role of laparoscopic distal gastrectomy in early gastric cancer has been well established, its application to advanced gastric cancer remains more technically and oncologically challenging. In contrast to early-stage disease, advanced gastric cancer typically requires more extensive lymph node dissection, at least D2 lymphadenectomy, and is frequently associated with bulky nodal disease and more complex anatomy. These factors raise legitimate concerns regarding the technical feasibility and oncologic adequacy of laparoscopic dissection, particularly in the suprapancreatic regions. In response to these concerns, several randomized controlled trials have been conducted to evaluate the safety and oncologic validity of laparoscopic gastrectomy in patients with advanced gastric cancer.

The KLASS-02 trial was conducted as a phase III randomized controlled trial conducted across 14 high-volume centers in Korea, designed to address concerns regarding the technical adequacy of laparoscopic D2 lymphadenectomy [16]. The trial enrolled 1050 patients with cT2-T4a gastric cancer, randomized to LDG or ODG. The number of retrieved lymph nodes were comparable between groups (46.8 in LDG vs. 47.4 in ODG, *p* = 0.451) [17]. The primary endpoint, 3-year relapse-free survival, was comparable between LDG and ODG (80.3% vs. 81.3%, *p* = 0.726), confirming non-inferiority of the laparoscopic approach [18,19]. Recurrence patterns were also comparable, supporting the oncologic adequacy of laparoscopic D2 dissection. Notably, the rate of late postoperative complications was significantly lower in the laparoscopic group (6.5% vs. 11.0%, *p* = 0.01), highlighting a sustained long-term morbidity benefit of the minimally invasive approach. These findings indicate that LDG for locally advanced gastric cancer achieves comparable oncologic outcomes with a more favorable long-term morbidity profile.

A Japanese multicenter, non-inferiority randomized trial JLSSG0901 provided further evidence for the oncologic safety of laparoscopic gastrectomy in locally advanced gastric cancer [20,21]. This trial enrolled 502 patients with cT2-T4a gastric cancer across 37 institutions in Japan. The primary end point, 5-year relapse-free survival, was comparable between the two groups (75.7% in LDG vs. 73.9% in ODG; HR 0.96, 90% CI 0.72–1.26), confirming non-inferiority of the laparoscopic approach. Overall survival and recurrence patterns were also similar, with a comparable number of lymph nodes. Perioperative outcomes were largely comparable, although laparoscopic surgery was associated with lower blood loss and longer operative time. Postoperative complication rates, including severe complications, were similar between groups, indicating that laparoscopic distal gastrectomy with D2 lymphadenectomy achieves noninferior oncologic outcomes without compromising surgical safety.

Finally, the Chinese CLASS-01 trial provided large-scale evidence supporting noninferior oncologic outcomes of LDG compared with ODG in patients with locally advanced gastric cancer [22,23]. This multicenter randomized noninferiority trial enrolled 1056 patients across 14 centers. In the updated 5-year analysis, overall survival was comparable between the two groups (72.6% vs. 76.3%; HR = 1.17; 95% CI, 0.93–1.48), with no stage-specific survival differences observed. Lymph node retrieval and perioperative morbidity were also similar, supporting the oncologic validity of laparoscopic D2 gastrectomy in a large and diverse population.

Extending these findings to more advanced disease, the UMC-UPPERGI-01 trial from Vietnam focused specifically on patients with serosa-invasive (cT4a) gastric cancer [24]. Postoperative morbidity and major complications were comparable between LDG and ODG (22.1% vs. 21.2% and 2.9% vs. 3.8%, respectively), with similar lymph node retrieval (median, 32.5 vs. 33). However, because long-term oncologic outcomes are still under follow-up, these findings should currently be interpreted as short-term safety data for cT4a disease rather than definitive survival evidence.

Across multiple randomized trials, laparoscopic distal gastrectomy has demonstrated noninferior oncologic outcomes compared with open surgery in selected patients with locally advanced gastric cancer, despite the technical demands of D2 lymphadenectomy, with comparable lymph node retrieval, survival, and recurrence patterns. These results should be interpreted in the context of trial eligibility criteria, as patients with bulky nodal disease were generally excluded. In contrast to trials conducted in other countries where postoperative morbidity was comparable between two approaches, a consistent reduction in postoperative complications has been observed in the KLASS trials, including both early-stage and advanced disease settings. While multiple factors may contribute to this difference, it may be related to structured quality-control processes, surgeon credentialing, and standardized operative techniques implemented within the KLASS trial framework. These considerations will be discussed further in subsequent sections. Taken together, current evidence most strongly supports laparoscopic distal gastrectomy for selected patients with resectable, non-bulky, locally advanced distal gastric cancer, particularly when performed by experienced surgeons in centers with established quality-control systems (Table 1).

### 2.3. Laparoscopic Total Gastrectomy and Beyond

Laparoscopic total gastrectomy (LTG) represents one of the most technically demanding procedures in minimally invasive gastric surgery, primarily due to the complexity of esophagojejunostomy, the risk of anastomotic leakage, and the need for extensive suprapancreatic lymph node dissection. Consequently, early concerns focused not on oncologic efficacy but on whether LTG could be performed safely and reproducibly.

The KLASS-03 trial provided early prospective evidence supporting the technical feasibility of LTG [25]. Conducted as a multicenter phase II study in high-volume Korean centers with strict surgeon credentialing and quality control, it demonstrated acceptable short-term outcomes, with a postoperative morbidity rate of 20.6%, a major complication (Clavien–Dindo grade ≥ III) rate of 9.4%, a mortality rate of 0.6%, and a minimal conversion rate to open surgery. These findings confirmed that LTG can be performed safely and reproducibly in experienced hands, establishing a foundation for subsequent comparative trials.

Building on this feasibility, the CLASS-02 trial provided randomized data on the short-term safety of LTG. This multicenter, open-label, noninferiority trial conducted across 14 high-volume centers in China enrolled 214 patients with clinical stage I gastric cancer and compared laparoscopic and open total gastrectomy [26]. Postoperative complication rates were comparable between the two groups (19.1% vs. 20.2%), with no significant differences in major complications or mortality [27]. Lymph node retrieval and R0 resection rates were also similar, suggesting that laparoscopic surgery did not compromise surgical radicality. Long-term follow-up results have recently been reported, demonstrating comparable 5-year overall survival (93.3% vs. 94.5%, *p* = 0.722) and disease-free survival (92.4% vs. 93.6%, *p* = 0.723) between the laparoscopic and open groups [28]. While limited to early-stage disease, these findings extend the evidence for LTG beyond short-term outcomes and support its oncologic adequacy.

While CLASS-02 validated LTG in early-stage disease, the STOMACH trial extended these findings to a more complex clinical setting, including patients with advanced gastric cancer undergoing neoadjuvant chemotherapy [29]. This European multicenter randomized trial demonstrated comparable oncologic outcomes between minimally invasive and open total gastrectomy, with similar lymph node retrieval (40.7 vs. 44.3, *p* = 0.209) and 3-year overall survival (46.8% vs. 57.1%, *p* = 0.186), without an increase in major postoperative complications. Postoperative mortality occurred only in the open surgery group (2 cases, 4.1%), with no deaths observed in the laparoscopic group [30]. These findings suggest that minimally invasive total gastrectomy can be safely applied in the context of advanced disease and multimodal treatment.

The LOGICA trial further evaluated the generalizability of minimally invasive gastrectomy in Western clinical practice, where patients more often present with advanced disease and receive perioperative chemotherapy [31]. This multicenter randomized controlled trial conducted in the Netherlands compared laparoscopic and open surgery, including both distal and total gastrectomy, with total gastrectomy accounting for approximately 40% of cases, and showed no significant differences in morbidity, major complications, R0 resection, lymph node retrieval, or hospital stay, although operative time was longer and blood loss was reduced in the laparoscopic group [32]. Notably, these findings were consistent across subgroup analyses, including procedure type (DG vs. TG) and disease stage (EGC vs. AGC). One-year overall survival was also comparable between groups (76% vs. 78%, *p* = 0.74), although long-term survival outcomes are still under follow-up. Postoperative mortality (approximately 4% vs. 7% in the laparoscopic and open groups, respectively) was higher than that reported in East Asian randomized trials, although no difference between approaches was observed, with a trend toward lower mortality in the laparoscopic group. These results support the safe implementation of minimally invasive gastrectomy, including total gastrectomy, in Western practice under appropriate training and quality control.

Western trials are particularly informative because they include clinical settings in which higher BMI, gastroesophageal junction tumors, and perioperative chemotherapy may be more frequently encountered. These factors can increase operative complexity by limiting exposure, increasing the need for technically demanding reconstruction, or causing treatment-related tissue fibrosis. Nevertheless, the STOMACH and LOGICA trials suggest that minimally invasive gastrectomy can be safely evaluated and implemented in appropriately selected patients within experienced and structured surgical programs, and may be particularly relevant when postoperative recovery and continuation of multimodal treatment are considered.

Taken together, these studies indicate a stepwise expansion of minimally invasive total gastrectomy—from feasibility to randomized validation and extension to multimodal Western settings—supporting its broader adoption beyond high-volume East Asian centers. Ongoing randomized trials, such as the KLASS-06 study comparing laparoscopic and open total gastrectomy in advanced gastric cancer, are expected to further clarify its role in this context [33]. A summary of the key clinical studies evaluating laparoscopic versus open total gastrectomy is provided in Table 2.

Reflecting the accumulating evidence, current clinical practice guidelines show a generally consistent approach to laparoscopic gastrectomy, although the strength of recommendation varies by region. The Japanese guidelines strongly endorse laparoscopic distal gastrectomy for early-stage disease but remain conservative regarding laparoscopic total gastrectomy and advanced-stage tumors, citing limited long-term data and technical complexity [34]. In contrast, the Korean guidelines more explicitly accept laparoscopic gastrectomy as an appropriate option not only for early but also for selected locally advanced gastric cancer, based on randomized evidence demonstrating oncologic equivalence and acceptable morbidity [35]. Western guidelines, including those from ESMO and the NCCN, acknowledge the growing evidence but recommend its selective use in experienced centers, emphasizing careful patient selection and surgical expertise, particularly in the setting of advanced disease, neoadjuvant therapy, or technically complex tumors [36,37].

Collectively, these guidelines indicate a global trend toward broader acceptance of laparoscopic gastrectomy, with strong agreement in early-stage disease and increasing use in more advanced settings. However, further evidence—especially in advanced disease and multimodal treatment—is still needed to support more consistent recommendations across regions.

## 3. Beyond Standard Laparoscopy

### 3.1. Robotic Gastrectomy

Robotic gastrectomy has emerged as a prominent extension of minimally invasive gastric cancer surgery, driven by the limitations of conventional laparoscopy in technically demanding procedures. The robotic platform offers several theoretical and practical advantages, including three-dimensional high-definition visualization, tremor elimination, articulated instruments with increased degrees of freedom, and improved surgeon ergonomics, which are particularly advantageous for complex tasks such as suprapancreatic lymph node dissection, intracorporeal reconstruction, and minimally invasive total gastrectomy [38].

Early retrospective and single-center series demonstrated the technical feasibility and safety of robotic gastrectomy, with outcomes comparable to laparoscopic surgery, establishing it as a viable minimally invasive alternative. Subsequently, a substantial body of retrospective and propensity score-matched studies have compared robotic and laparoscopic gastrectomy across different settings [39,40,41,42,43,44,45]. Most reports show reduced intraoperative blood loss and, in some series, lower postoperative morbidity with robotic surgery, while operative time and costs tend to be higher. Long-term oncologic outcomes, including overall and disease-free survival, are generally comparable between approaches. However, these observational data, despite suggesting perioperative advantages, do not demonstrate a clear oncologic benefit over conventional laparoscopy.

To date, only a limited number of randomized trials have directly compared robotic and laparoscopic gastrectomy. A phase III randomized trial from Japan evaluated short-term perioperative outcomes of robotic versus laparoscopic gastrectomy. The trial did not meet its primary endpoint, as the incidence of intra-abdominal infectious complications was not significantly different between the two groups (6.2% vs. 8.5%, *p* = 0.50) [46]. However, robotic gastrectomy was associated with lower overall postoperative complications (8.8% vs. 19.7%, *p* = 0.02) and lower major complications (5.3% vs. 16.2%, *p* = 0.01). These findings suggest potential short-term perioperative benefits, but do not provide evidence on long-term oncologic outcomes compared with conventional laparoscopy.

A phase II randomized non-inferiority trial from China compared robotic and laparoscopic distal gastrectomy in patients with resectable gastric cancer, with 3-year disease-free survival as the primary endpoint. The study reported higher 3-year disease-free survival in the robotic group than in the laparoscopic group (85.8% vs. 73.2%, *p* = 0.011), meeting the predefined non-inferiority criterion [47]. Short-term outcomes also demonstrated lower postoperative morbidity in the robotic group (9.2% vs. 17.6%, *p* = 0.039), along with faster recovery, milder inflammatory responses, and higher retrieved extraperigastric lymph nodes [48]. Because comparable survival advantages have not been consistently observed in other randomized studies, this finding should be interpreted cautiously and should not be viewed as evidence of an inherent oncologic advantage of the robotic platform. A summary of the randomized trials comparing robotic and laparoscopic gastrectomy is provided in Table 3.

Robotic gastrectomy has a learning curve that may be less steep than advanced laparoscopic procedures, particularly for complex operations such as total gastrectomy and suprapancreatic lymphadenectomy [49]. Nevertheless, outcomes remain highly dependent on surgeon experience, institutional volume, and structured training programs. In addition, cost remains a major barrier to the wider adoption of robotic gastrectomy. Recent economic evaluations suggest that potential perioperative or quality-of-life benefits may be offset by higher direct procedural costs and uncertain cost-effectiveness compared with conventional or articulating-instrument laparoscopy [50,51]. Therefore, routine use should require evidence of clinical value and sustainable resource utilization, not technical feasibility alone.

Taken together, robotic gastrectomy represents a safe and technically advantageous approach in selected settings but consistent superiority over conventional laparoscopy in morbidity, oncologic outcomes, or cost-effectiveness has not been established. It should therefore be considered a complementary modality, with its role likely to evolve as further evidence accumulates.

### 3.2. Function-Preserving Surgery

As minimally invasive gastrectomy has become established, laparoscopic function-preserving procedures have emerged as an extension of this approach, aiming to reduce treatment-related burden not only through less invasive access but also through limited resection and tailored reconstruction to preserve postoperative function. Function-preserving gastrectomy, particularly pylorus-preserving gastrectomy (PPG) and proximal gastrectomy (PG), has emerged as an important strategy in the management of early gastric cancer [52]. These procedures require strict selection of early-stage tumors with low nodal risk and appropriate location: PPG is generally considered for middle-third tumors sufficiently distant from the pylorus, whereas PG is considered for upper-third tumors when an adequate distal remnant stomach can be preserved.

Early retrospective and single-center studies have provided distinct insights into PPG and PG. These studies have consistently demonstrated that PPG achieves oncologic outcomes comparable to distal gastrectomy, while offering functional advantages such as reduced incidence of dumping syndrome, bile reflux, and gallstone formation, along with better preservation of hemoglobin, serum protein levels, and body weight [53,54,55]. However, PPG has also been associated with a higher incidence of delayed gastric emptying, reflecting a procedure-specific trade-off.

Similarly, PG has been shown to have comparable survival outcomes to total gastrectomy, while providing improved nutritional and hematologic profiles, including reduced vitamin B12 deficiency, better maintenance of hemoglobin levels, and less body weight loss [56,57,58]. Postoperative outcomes after PG depend substantially on the reconstruction method. Anti-reflux esophagogastrostomy techniques, such as the double-flap method, may reduce reflux but are technically demanding and may carry a risk of anastomotic stenosis, whereas double-tract reconstruction has also shown favorable reflux-related outcomes. However, comparative evidence remains insufficient to define the optimal reconstruction method after PG.

These findings have subsequently been evaluated in multicenter randomized trials. The KLASS-04 trial demonstrated that PPG achieves comparable oncologic outcomes to distal gastrectomy, while offering selected functional advantages, albeit with an increased risk of delayed gastric emptying [59,60]. Similarly, the KLASS-05 trial showed that PG with double-tract reconstruction provides comparable oncologic safety to total gastrectomy, with favorable nutritional outcomes, including reduced vitamin B12 supplementation requirements and improved postoperative functional status [61,62].

Taken together, function-preserving gastrectomy represents a meaningful evolution of minimally invasive gastric cancer surgery, shifting the focus from short-term recovery to long-term functional outcomes and quality of life. However, its application remains largely concentrated in high-volume centers in Korea and Japan, and careful patient selection, standardized reconstruction techniques, and adequate surgical expertise are essential to maximize its benefits.

### 3.3. Reduced-Port Surgery

Beyond platform-based advancements such as robotic surgery, reduced-port laparoscopic gastrectomy has emerged as a further refinement of minimally invasive gastric cancer surgery, aiming to minimize abdominal wall trauma and improve cosmetic outcomes while preserving oncologic radicality. Although early single-institution series suggested technical feasibility, widespread adoption was limited by ergonomic constraints and steep learning curves.

More recently, higher-level evidence has begun to emerge. A single-center randomized controlled trial in Japan comparing single-port and multi-port laparoscopic distal gastrectomy for stage I disease demonstrated comparable long-term quality-of-life outcomes, with selected improvements in postoperative pain, inflammatory markers, and weight loss in the single-port group [63]. Nationwide Korean data have likewise shown comparable complication rates in reduced-port procedures, although their adoption has remained selective even in high-volume centers [64].

Randomized multicenter validation was provided by the KLASS-12 trial, which confirmed non-inferiority of reduced-port distal gastrectomy for 30-day postoperative complications in early gastric cancer, with comparable lymph node retrieval, conversion rates, and length of stay, and slightly lower pain scores at postoperative day five [65]. Rigorous surgeon credentialing and video-based quality control were integral to the trial design, emphasizing the importance of experience and institutional systems.

Parallel developments have also been observed in robotic platforms. Nationwide Korean registry data in KLASS-13 study demonstrated that reduced-port robotic gastrectomy was associated with a shorter hospital stay and comparable major complication rates compared with conventional laparoscopic surgery, with a very low conversion rate, supporting its feasibility in experienced centers [66].

**Figure 1:**
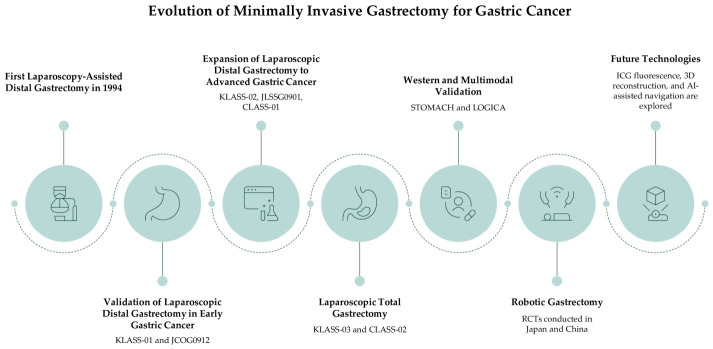
Evolution of Minimally Invasive Gastrectomy for Gastric Cancer. Key milestones include the first laparoscopy-assisted distal gastrectomy, randomized validation in early and selected advanced gastric cancer, laparoscopic total gastrectomy, Western multimodal validation, robotic gastrectomy, and emerging adjunct technologies such as ICG fluorescence imaging, three-dimensional reconstruction, and artificial intelligence-assisted navigation.

Recent studies indicate ongoing technological evolution of reduced-port platforms, although most evidence remains derived from experienced institutions with established minimally invasive gastrectomy programs [67]. At present, reduced-port gastrectomy remains a technically demanding and selective approach, with evidence largely limited to highly selected patients undergoing early-stage distal gastrectomy. Evidence for advanced gastric cancer or total gastrectomy is insufficient. Although cosmetic benefits and modest improvements in pain or inflammatory response have been reported, these should not outweigh the need for oncologic safety, adequate lymphadenectomy, reliable reconstruction, and reproducible surgical quality. Therefore, it should be regarded as an evolving technical refinement rather than a new standard. The stepwise development of minimally invasive gastrectomy described in the preceding sections is summarized in Figure 1.

## 4. MIS for Reducing Surgical Morbidity

Postoperative morbidity after minimally invasive gastrectomy varies across trials, despite generally favorable perioperative recovery and comparable oncologic outcomes in selected patients. This variability may partly reflect differences in patient-level risk factors, such as age, comorbidity, frailty, tumor extent, and perioperative therapy. It may also reflect differences in the surgical environment in which minimally invasive gastrectomy is performed.

Among contemporary randomized trials, the KLASS series represents a notable exception in which minimally invasive surgery was associated with a statistically significant reduction in postoperative complications. This finding should be interpreted in the context of the highly structured surgical environment of these trials, including concentration of surgical expertise and rigorous quality-assurance processes.

Population-based studies provide strong evidence that surgeon and hospital volume exert major influences on postoperative outcomes after gastrectomy. Japanese nationwide registry analyses of more than 70,000 total gastrectomies demonstrated stepwise declines in operative mortality with increasing institutional and surgeon volume [68], while the CRITICS trial cohort similarly showed superior outcomes in high-volume hospitals [69]. A recent meta-analysis encompassing nearly 600,000 patients confirmed that postoperative mortality was approximately 35% lower in institutions with substantial gastrectomy experience and plateaued only when annual volumes approached 100 gastrectomies [70].

In Korea, this volume–outcome relationship is particularly relevant because gastric cancer surgery is highly centralized. In a nationwide Korean Gastric Cancer Association analysis, almost half of all gastrectomies were performed in a small number of specialized centers performing more than 500 cases annually, and minimally invasive surgery accounted for the majority of procedures [71]. Severe complications after MIS declined stepwise as institutional volume increased, whereas no similar gradient was observed for open surgery. Although hospital volume lost independent significance after multivariable adjustment, these findings illustrate how concentration of expertise and procedural experience may provide important context for interpreting morbidity outcomes in Korean randomized trials.

Beyond crude volume effects, structured quality-assurance frameworks may also contribute to consistent perioperative outcomes. The KLASS-02 program incorporated rigorous surgeon credentialing and quality-control, including central review of unedited videos of suprapancreatic D2 lymphadenectomy using a structured checklist before patient enrollment [72]. Subsequent analyses demonstrated that higher quality-control scores were independently associated with lower blood loss, fewer major complications, and shorter hospital stay, suggesting that intensive procedural governance, supporting the relevance of procedural standardization and performance monitoring when interpreting morbidity outcomes in the KLASS series [73].

Similar attention to quality assurance has been reported in Western randomized trials. In the LOGICA study, surgical teams were required to complete hands-on proctoring before trial initiation, meet minimum institutional volume thresholds, and submit operative videos for central review. During the trial, prospective quality control was implemented through standardized intraoperative photography of suprapancreatic nodal stations, with weekly feedback provided to participating centers [74]. These measures coincided with consistently high radicality (95% R0 resection) and nodal yields (median 29 lymph nodes; ≥15 nodes in 95% of patients), supporting the interpretation that structured quality-control frameworks can stabilize surgical performance across institutions.

In summary, reductions in morbidity after minimally invasive gastrectomy are not attributable to the surgical approach alone, but reflect the combined effects of technical precision and system-level factors such as patient selection, centralization, surgeon experience, and structured quality control. Consistent observations across both Eastern and Western trials indicate that optimal outcomes depend not only on technical innovation but also on the environments in which surgery is delivered.

## 5. Suggestions for Safe Initiation of MIS

Translation of trial-level success in minimally invasive gastrectomy into routine clinical practice requires more than access to advanced surgical platforms. Accumulating evidence indicates that safe implementation depends on coordinated institutional programs encompassing patient selection, surgeon training, procedural standardization, quality monitoring, and perioperative infrastructure [75]. These elements may be considered as a practical framework for institutions initiating or expanding MIS gastrectomy programs and are summarized in Table 4.

First, patient selection should be stepwise and risk-adapted. During the early phase of program development, careful patient selection is essential to mitigate perioperative risk while teams move through learning curves. Initial cases should preferentially involve technically straightforward scenarios, such as early-stage distal tumors in non-obese patients without bulky nodal disease, adjacent organ involvement, or prior extensive abdominal surgery. Expansion to more complex indications—including total gastrectomy, D2 lymphadenectomy for locally advanced disease, and patients treated with neoadjuvant chemotherapy—should occur in a stepwise fashion as operative proficiency and multidisciplinary coordination mature. Such phased implementation mirrors the trajectory observed in major randomized programs, in which feasibility in selected populations preceded broader indications.

Second, case accumulation and procedure-specific learning curves should be recognized before expanding indications. Early Korean and Japanese series consistently demonstrated that laparoscopic distal gastrectomy demands approximately 50–60 cases before operative time stabilizes, lymph node retrieval becomes consistent, and complication rates decline, as summarized in multiple institutional learning-curve analyses [76]. Meanwhile, a cumulative-sum analysis indicated that approximately 100 cases are required to achieve proficiency in laparoscopic total gastrectomy, based on stabilization of operative performance [77]. Importantly, subsequent studies have shown that increasing surgeon experience and overcoming the learning curve are associated with improvements in oncologic quality and long-term survival [78,79]. These thresholds should be interpreted as approximate benchmarks rather than fixed requirements, because the learning curve varies according to procedure complexity, prior experience, team stability, and institutional support.

Third, structured training and credentialing are essential components of safe dissemination. Institutional educational systems, standardized operative protocols, stable operative teams, and mentorship have repeatedly been shown to shorten the time to proficiency. Tokunaga and colleagues reported that structured educational programs and standardized techniques enabled trainees to perform laparoscopic gastrectomy safely once adequate assisting experience had been accrued [80]. These findings support current trial designs and guideline recommendations that emphasize credentialing, proctorship, and continuous audit for safe dissemination. However, not all institutions can independently reproduce the credentialing and quality-control systems used in high-volume trials. In lower-volume centers, safer implementation may require external support, including regional proctorship, shared video review, referral of complex cases to experienced centers, and participation in multicenter audit networks.

Fourth, quality monitoring should include both composite outcomes and core audit metrics. Rather than evaluating single outcomes such as complications or mortality alone, recent approaches assess overall surgical quality using composite measures that integrate multiple perioperative outcomes. The concept of “textbook outcome,” integrating oncologic adequacy (R0 resection and sufficient lymph node harvest), absence of major complications, avoidance of reintervention or mortality, and timely discharge, has been proposed as a clinically meaningful benchmark for safe dissemination of complex oncologic procedures [81]. Registry-based studies in gastric cancer surgery have demonstrated that achievement of textbook outcome is strongly associated with institutional volume, surgeon experience, and structured perioperative pathways, reinforcing its utility as both a quality-monitoring tool and an implementation target during program maturation [82]. Incorporating such composite metrics into routine audit frameworks allows early identification of performance gaps and provides an objective scaffold for continuous improvement as MIS programs expand.

Fifth, operative standardization is required to maintain oncologic quality. In laparoscopic gastrectomy, consistent D2 lymphadenectomy is important to ensure reliable oncologic outcomes. Oncologic equivalence in minimally invasive gastrectomy depends on consistent suprapancreatic nodal dissection, and variation in technique during the early phase can lead to inadequate lymph node retrieval. To address this, multicenter trials and educational programs have adopted standardized definitions of nodal stations, stepwise dissection sequences, and intraoperative documentation using photography or video review. These measures have been associated with consistent lymph node yields and stable R0 resection rates across institutions, indicating that technical standardization is necessary in addition to individual surgical skill.

Sixth, institutional and multidisciplinary support should be established before broad expansion. Health-system organization further modulates the safety of MIS implementation. Gastrectomy performed in experienced centers is consistently associated with lower morbidity and mortality. Centralization allows surgeons to accumulate experience, supports dedicated operative teams and perioperative pathways, and enables structured training and complication management. It also provides sufficient case volume to progress through the learning curve within a reasonable timeframe, which is difficult to achieve in low-volume settings. Regular case discussion in tumor boards helps ensure appropriate patient selection and coordination of perioperative treatment. Close collaboration with anesthesiology, radiology, pathology, and enhanced-recovery teams improves perioperative stability and supports early recognition and management of complications. Such coordinated care has been a consistent feature of high-performing centers and is essential for maintaining stable surgical outcomes.

## 6. Conclusions and Future Directions

Minimally invasive gastrectomy has evolved from a technically demanding alternative to an established surgical approach with oncologic outcomes comparable to open surgery. Although open gastrectomy with adequate lymphadenectomy remains an important reference standard, randomized trials and large-scale studies support the safety and feasibility across early and selected advanced gastric cancer, including total gastrectomy. Its application should be individualized according to clinical stage, tumor location, planned perioperative treatment, and multidisciplinary tumor board discussion. However, these results have been largely achieved in institutions with substantial case experience with structured training, standardized techniques, and robust quality-control systems, highlighting a gap between trial outcomes and real-world implementation.

Future research should therefore shift from comparing surgical approaches to identifying strategies that enable consistent and reproducible outcomes across diverse clinical settings. In particular, randomized trials are needed in more complex clinical scenarios—such as patients receiving neoadjuvant therapy, those with advanced disease, and higher-risk populations—where clinically meaningful endpoints extend beyond short-term morbidity to include composite outcomes, functional recovery, and completion of multimodal treatment.

Technological advances, including robotic platforms, reduced-port techniques, and image-guided surgery, may further improve surgical precision. Emerging intraoperative adjuncts may also play an increasing role in minimally invasive gastric cancer surgery. Indocyanine green fluorescence imaging has been explored for lymphatic mapping, lymph node navigation, and assessment of anastomotic perfusion, while three-dimensional reconstruction and artificial intelligence-based platforms may assist surgical planning and intraoperative navigation. However, their evaluation should incorporate learning curves, cost-effectiveness, and institutional readiness, rather than focusing solely on technical feasibility.

Equally important are system-level factors. Harmonization of training and credentialing, supported by multinational registries, video-based quality control, and standardized certification programs, will be essential to reduce variability and ensure safe dissemination of complex procedures. In parallel, health-system research is needed to define optimal volume thresholds and referral pathways, particularly in low-incidence settings, where centralization may be required to achieve acceptable outcomes.

Finally, economic sustainability will remain a key consideration. Cost–utility analyses incorporating long-term oncologic outcomes, complication-related costs, and quality-of-life measures are needed to define the appropriate role of advanced minimally invasive approaches within different healthcare systems.

Taken together, current evidence suggests that the success of minimally invasive gastrectomy is determined not only by technical innovation, but by the systems that support its implementation. Future progress will therefore depend less on further refinement of surgical techniques than on the ability to standardize training, ensure quality control, and integrate multidisciplinary care across institutions.

## Figures and Tables

**Table 1:** Randomized controlled trials comparing laparoscopic distal gastrectomy and open distal gastrectomy.

Trial	Country	N	Stage	Primary Endpoint	Main Results
**Early Gastric Cancer**					
KLASS01	Korea	1416	cStage I	5Y OS	Comparable 5Y OS of LDG vs. ODG (94.2% vs. 93.3%, *p* = 0.64) Comparable cancer-specific survival of LDG vs. ODG (97.1% vs. 97.2%, *p* = 0.91) Lower overall complication rate in LDG (13.0% vs. 19.9%, *p* = 0.001)
JCOG0912	Japan	921	cStage I	5y RFS	Comparable 5Y RFS of LDG vs. ODG (94.0% vs. 95.1%, (HR, 0.84 [90% CI, 0.56–1.27]), *p* = 0.0075) Comparable in-hospital grade 3–4 surgical complications (3.3% in LDG vs. 3.7% in ODG, *p* = 0.72)
**Advanced gastric cancer**					
KLASS02	Korea	1050	cT2-T4a cN0 or Limited perigastric LN	3Y RFS	Comparable 3Y RFS of LDG to ODG (80.3% vs. 81.3%, *p* = 0.726) Comparable 5Y OS of LDG to ODG (88.9% vs. 88.7%, *p* = 0.30) Lower early complication rate of LDG (4.7% vs. 9.5%, *p* = 0.0038) Lower late complication rate of LDG (6.5% vs. 11.1%, *p* = 0.01)
JLSSG0901	Japan	502	cT2-T4a cN0–2, excluding bulky LN	5Y RFS	Comparable 5Y RFS of LDG to ODG (75.7% vs. 73.9%) Comparable 5Y OS of LDG to ODG (81.7% vs. 79.8%) Comparable overall postoperative complications (3.1% vs. 4.7%, *p* = 0.473)
CLASS01	China	1056	cT1-T4a cN0-3, excluding bulky LN	3Y DFS	Comparable 3Y DFS of LDG to ODG (76.5% vs. 77.8%) Comparable 3Y OS of LDG to ODG (83.1% vs. 85.2%) Comparable postoperative complication rate of LDG to ODG (15.2% vs. 12.9%, *p* = 0.845)
UMC- UPPERGI01	Vietnam	208	cT4aN0-3, excluding bulky LN	3Y DFS	Comparable overall postoperative complications (22.1% vs. 21.2%; *p* = 0.87) Comparable mortality rate (1.9% vs. 1.0%, *p* > 0.99) Ongoing follow-up for survival outcomes

Abbreviations: LDG, laparoscopic distal gastrectomy; ODG, open distal gastrectomy; OS, overall survival; DFS, disease-free survival; RFS, relapse-free survival; LN, lymph node.

**Table 2:** Randomized and prospective studies evaluating laparoscopic total gastrectomy for gastric cancer.

Trial	Country	N	Stage	Primary Endpoint	Secondary Endpoints	Main Results
KLASS03 (Single-arm)	Korea	170	cStage I	Postoperative morbidity and mortality		Postoperative morbidity rate 20.6%, Major complication rate 9.4% Postoperative mortality rate 0.6%
CLASS02	China	214	cStage I	Postoperative morbidity and mortality	5Y OS and DFS rates	Comparable 5Y OS of LTG to OTG (93.3% vs. 94.5%, *p* = 0.722) Comparable 5Y DFS of LTG to OTG (92.4% vs. 93.6%, *p* = 0.723) Comparable overall postoperative complication rate (18.1% vs. 17.4%)
STOMACH	Europe	96	cT1-4a, N0-3 periop chemotherapy	Quality of oncological resection	3Y OS	Comparable 3Y OS of LTG to OTG (46.8% vs. 57.1%, *p* = 0.186) Comparable number of resected lymph nodes (40.7 vs. 44.3, *p* = 0.209)
LOGICA (Included both DG and TG)	Netherlands	227 (TG = 91)	cT1-4aN0-3bM0	Hospital stay days	postoperative complications and mortality, overall survival, quality of life, etc.	Comparable 1Y OS of LTG vs. OTG (76% vs. 78%, *p* = 0.74) Comparable postoperative complications, in-hospital mortality, hospital stay

Abbreviations: LTG, laparoscopic total gastrectomy; OTG, open total gastrectomy; DG, distal gastrectomy; TG, total gastrectomy; OS, overall survival; DFS, disease-free survival.

**Table 3:** Randomized controlled trials comparing robotic and laparoscopic gastrectomy for gastric cancer.

Trial	Country	N	Procedure Types	Stages	Main Results
Ojima et al.	Japan	241	DG, TG, PG	Resectable GC (cStage I–III)	No difference in infectious complications Lower overall complication rates in Robotic group (8.8% vs. 19.7%, *p* = 0.02) Lower major complication rates in Robotic group (5.3% vs. 16.2%, *p* = 0.01)
Lu et al.	China	283	Only DG	Resectable GC (cStage I–III)	Lower overall complication rate in Robotic group (9.2% vs. 17.6%, 0.039) Higher 3-year disease-free survival in Robotic group (85.8% vs. 73.2%, *p* = 0.011)

Abbreviations: PG, proximal gastrectomy; DG, distal gastrectomy; TG, total gastrectomy; GC, gastric cancer.

**Table 4:** Key considerations for safe implementation and quality monitoring of minimally invasive gastrectomy.

Checklist	Key Elements
Stepwise patient selection	Begin with technically favorable cases, such as early-stage distal tumors Gradual expansion to more advanced disease, total gastrectomy, or neoadjuvant chemotherapy treated patients
Learning curve monitoring	Accumulate procedure-specific experience
Structured training and credentialing	Standardized education; mentorship; proctorship; video review; shared audit; external support or referral pathways for lower-volume centers
Quality monitoring	Textbook outcome measurements: R0 resection rate, lymph node yield, major complications, reoperation, ICU admission, 30-day mortality, length of stay, and readmission
Operative standardization	Standardized D2 lymphadenectomy; nodal station definitions; stepwise dissection; reconstruction protocol; clear conversion criteria; photo/video documentation
Institutional and multidisciplinary support	Centralization, dedicated operative teams, complication management system; referral pathway; multicenter audit network, tumor board

## Data Availability

No new data were created or analyzed in this study. Data sharing is not applicable.
